# Knockdown and overexpression of Unc-45b result in defective myofibril organization in skeletal muscles of zebrafish embryos

**DOI:** 10.1186/1471-2121-11-70

**Published:** 2010-09-17

**Authors:** Elena P Bernick, Pei-Jun Zhang, Shaojun Du

**Affiliations:** 1 University of Maryland School of Medicine Interdisciplinary Training Program in Muscle Biology, Baltimore, MD 21201, USA; 2 Department of Biochemistry and Molecular Biology, University of Maryland School of Medicine, Baltimore, MD 21201, USA; 3 Institute of Oceanology, Chinese Academy of Sciences, Qingdao, China

## Abstract

**Background:**

Unc-45 is a myosin chaperone and a Hsp90 co-chaperone that plays a key role in muscle development. Genetic and biochemical studies in *C. elegans *have demonstrated that Unc-45 facilitates the process of myosin folding and assembly in body wall muscles. Loss or overexpression of Unc-45 in *C. elegans *results in defective myofibril organization. In the zebrafish *Danio rerio*, *unc-45b*, a homolog of *C. elegans unc-45*, is expressed in both skeletal and cardiac muscles. Earlier studies indicate that mutation or knockdown of *unc-45b *expression in zebrafish results in a phenotype characterized by a loss of both thick and thin filament organization in skeletal and cardiac muscle. The effects of *unc-45b *knockdown on other sarcomeric structures and the phenotype of Unc-45b overexpression, however, are poorly understood in vertebrates.

**Results:**

Both knockdown and overexpression provide useful tools to study gene function during animal development. Using such methods, we characterized the role of Unc-45b in myofibril assembly of skeletal muscle in *Danio rerio*. We showed that, in addition to thick and thin filament defects, knockdown of *unc-45b *expression disrupted sarcomere organization in M-lines and Z-lines of skeletal muscles in zebrafish embryos. Western blotting analysis showed that myosin protein levels were significantly decreased in *unc-45b *knockdown embryos. Similarly, embryos overexpressing Unc-45b also exhibited severely disorganized myosin thick filaments. Disruption of thick filament organization by Unc-45b overexpression depends on the C-terminal UCS domain in Unc-45b required for interaction with myosin. Deletion of the C-terminal UCS domain abolished the disruptive activity of Unc-45b in myosin thick filament organization. In contrast, deletion of the N-terminal TPR domain required for binding with Hsp90α had no effect.

**Conclusion:**

Collectively, these studies indicate that the expression levels of Unc-45b must be precisely regulated to ensure normal myofibril organization. Loss or overexpression of Unc-45b leads to defective myofibril organization.

## Background

Sarcomeres, the repetitive intracellular units within striated muscle fibers, are the basic contractile unit in skeletal and cardiac muscles. Sarcomeres contain highly organized arrangements of proteins and are responsible for the contractile properties of muscles. Two major types of filaments- thick and thin- can be found within these complex sarcomeric structures. In addition, sarcomeres contain other major structures, including the Z-lines and M- lines, which provide structural support to the sarcomere. Myofibrillogenesis, the process of assembling myofibrillar proteins into a highly organized sarcomere, is pivotal for normal muscle function and motility. Defective sarcomere organization often leads to malfunction and diseases of skeletal and cardiac muscles, including muscular dystrophy and cardiomyopathy [1].

The major protein components of the thick and thin filaments are myosin and α-actin, respectively. Recent studies indicate that molecular chaperones play an important role in myosin folding and thick filament assembly [2-4]. Heat shock protein 90α (Hsp90α) and Unc-45 are key myosin chaperones that are specifically expressed in skeletal and cardiac muscles and play crucial roles in myofibrillogenesis [5-11]. Knockdown or mutation of Hsp90α1 results in poor myofibril organization, myosin degradation, and paralysis of zebrafish embryos [7,8]. Hsp90α associates with Unc-45, a myosin chaperone which was first characterized in the nematode *Caenorhabditis elegans*, to control myofibril assembly. The *C. elegans *Unc-45 mutants showed decreased body movement and disorganized myofilament arrays in body wall muscles [12].

While *C. elegans *and *Drosophila melanogaster *express one isoform of Unc-45, many vertebrates, including the zebrafish, express two isoforms [9,13]. These two isoforms are known as Unc-45a and Unc-45b. Unc-45a, also known as general cell Unc-45, is expressed in many tissues and has roles in smooth muscle myosin maturation and development of the aortic arches [14,15]. Unc-45b, on the other hand, has been identified as a muscle-specific isoform expressed in striated muscles [13]. Biochemical and functional studies have revealed that Unc-45b binds to the myosin motor domain and plays key roles in myosin folding and sarcomere assembly [3,15,16]. Unc-45b knockdown or mutation results in cardiac defects and paralysis of zebrafish embryos [10,11].

Previous studies in *C. elegans *demonstrated that the protein levels of Unc-45 are highly regulated by the ubiquitin/proteasome pathway [17]. A new E3/E4 complex, formed by CHN-1, the *C. elegans *ortholog of CHIP (carboxyl terminus of Hsc70-interacting protein), and UFD-2, an enzyme known to have ubiquitin-conjugating E4 activity in yeast, is necessary and sufficient to multiubiquitylate Unc-45 *in vitro *and directs Unc-45 degradation [17]. Overexpression of Unc-45 in *C. elegans *results in thick filament defects, decreased myosin expression, and mild paralysis [12]. The decrease in myosin expression is attributed to increased protein degradation through the ubiquitin/proteasome pathway [12]. A similar conserved pathway was identified in humans, which links Unc-45b degradation and myosin assembly [18]. Remarkably, mutations in human p97, an ubiquitin-selective chaperone, cause hereditary inclusion body myopathy, abrogate Unc-45 degradation and result in severely disorganized myofibrils [18,19].

Structurally, Unc-45b is comprised of three major domains: an N-terminal domain containing three tetratricopeptide repeats (TPR), a central domain, and a C-terminal UCS (Unc-45/CRO1/She4p) domain [20]. The TPR domain interacts with the MMEVD motif at the C-terminus of Hsp90 [16]. The UCS domain is required for interaction with myosin [11,16]. Although the function of the central domain remains unknown, it contains regions of limited homology to β-catenin, a key player in the Wnt signaling pathway [3].

While previous studies have contributed significantly to our understanding of the role of Unc-45 in myosin folding, several questions regarding Unc-45 structure and function remain unanswered. Is Unc-45b required for organization of other sarcomeric structures in addition to the thick and thin filaments? Does overexpression of Unc-45b disrupt myofibril organization in vertebrate skeletal muscles? Which protein domain is involved in the disruptive effect of Unc-45b when overexpressed? Addressing these questions may lead to increased understanding of Unc-45b function in muscle physiology and development that promote new approaches combating chronic muscular disorders.

This study attempts to answer these questions and better ascertain the role of Unc-45b in sarcomere assembly in zebrafish embryos. Our results indicate that knockdown of Unc-45b result in disruption of sarcomere organization and decreased levels of myosin protein expression. We further showed that overexpression of Unc-45b resulted in similar disruption of myosin thick filament organization. The disruption of thick filament organization by Unc-45b depended on the C-terminal UCS domain required for interaction with myosin. Deletion of the C-terminal UCS domain abolished the disruptive effect of Unc-45b overexpression on thick filament organization. Together, these studies indicate that the expression levels of Unc-45b must be precisely regulated to ensure normal myofibril organization during muscle development. Knockdown and overexpression of Unc-45b result in defective myofibril organization.

## Results

### 1. Knockdown of unc-45b resulted in decreased myosin and actin protein accumulation in zebrafish embryos

**Figure 1:**
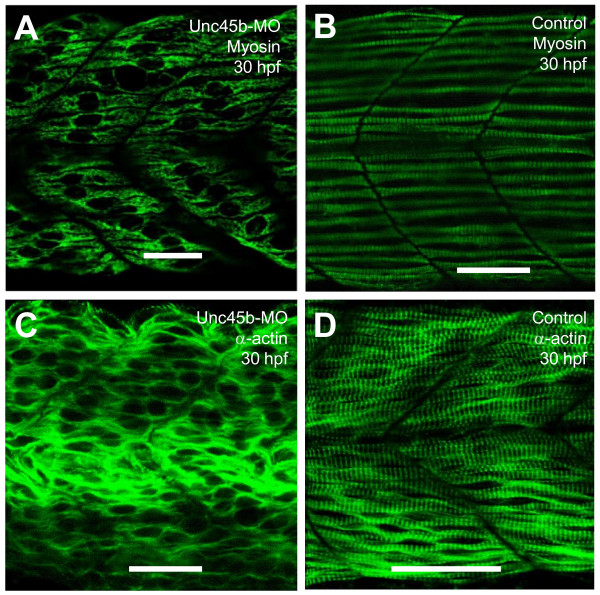
**Effects of *unc-45b *knockdown on thick and thin filament organization in skeletal muscles of zebrafish embryos**. **A, B**. Anti-MyHC antibody (F59) staining shows the organization of thick filaments in trunk slow muscles of uninjected control (B), or *unc-45b *knockdown (A) embryos at 30 hpf. **C, D**. Anti-α-actin antibody staining shows the organization of thin filaments in trunk muscles of uninjected control (D) or *unc-45b *knockdown (C) embryos at 30 hpf. Scale bar = 25 μm in A, B, C, D.

Unc-45b is a known myosin chaperone. It has been shown that knockdown or mutation of *unc-45b *in zebrafish embryos resulted in defective organization of myosin thick filaments and actin thin filaments [10,11]. However, it remains unclear whether the diminished organization of the thick and thin filaments was influenced by decreased myosin and actin protein accumulation or the inability of these proteins to assemble appropriately into thick and thin filaments. To assess the effect of *unc-45b *knockdown on myosin and actin protein accumulation, we knocked down *unc-45b *expression in zebrafish embryos and analyzed myosin and actin protein expression and localization. As expected, knockdown of *unc-45b *resulted in severe disruption of thick and thin filament organization in zebrafish embryos (Figure 1A, C), a phenotype similar to that previously reported [10,11].

**Figure 2:**
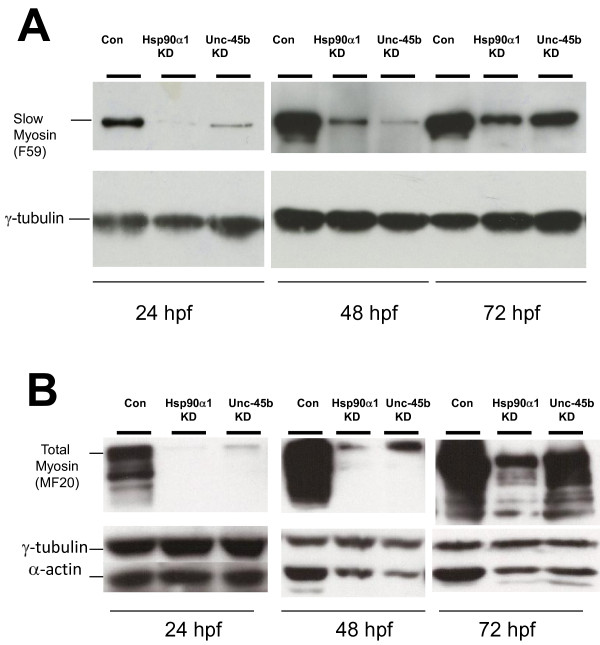
**Knockdown of Unc-45b or Hsp90α1 results in significant reduction of myosin and α-actin accumulation**. Western blot was performed using protein extracts from Unc-45b or Hsp90α1 knockdown, or uninjected control embryos at 24, 48 and 72 hpf using anti-myosin antibodies F59 (A) and MF20 (B) that recognize slow muscle myosin or general myosin proteins, respectively. The data show decreased levels of slow or total myosin proteins in Unc-45b and Hsp90α1 knockdown embryos at 24 and 48 hpf. A partial recovery of myosin expression was noted in 72 hpf embryos. Decreased levels of α-actin were also detected in the Unc-45b and Hsp90α1 knockdown embryos at 48 and 72 hpf. γ-tublin was used as loading control. Protein extract from five embryos each was used at 24 hpf. Protein extracts from three embryos were used at 48 and 72 hpf.

Western blotting was performed to determine the protein accumulation of myosin heavy chain and α-actin proteins in *unc-45b *knockdown embryos and compared with that from *hsp90α1 *knockdown. As illustrated in Figure 2, knockdown of *unc-45b *expression dramatically decreased the levels of myosin proteins in slow muscles (F59) and the total myosin content (MF20) in zebrafish embryos at 24 and 48 hours post-fertilization (hpf). This effect was very similar to that observed in *hsp90α*1 knockdown embryos (Figure 2, refs. [7,8]), consistent with the idea that Unc-45b works together with Hsp90α to control myosin folding and assembly [3]. By 72 hpf, a partial recovery of myosin expression was observed in *unc-45b *or *hsp90α1 *knockdown embryos (Figure 2), presumably due to the reduced levels of morpholino oligos from dilution and degradation by 72 hpf. Interestingly, α-actin protein levels appeared to be reduced in *unc-45b *or *hsp90α1 *knockdown embryos at 48 and 72 hpf (Figure 2B). This could be due to a secondary effect from disruption of myosin folding and assembly. Collectively, these data suggest that Unc-45b is required for myosin and actin protein accumulation and normal assembly into thick and thin filaments.

### 2. Unc-45b knockdown resulted in little or no sarcomeric localization of M- line protein in zebrafish embryos

**Figure 3:**
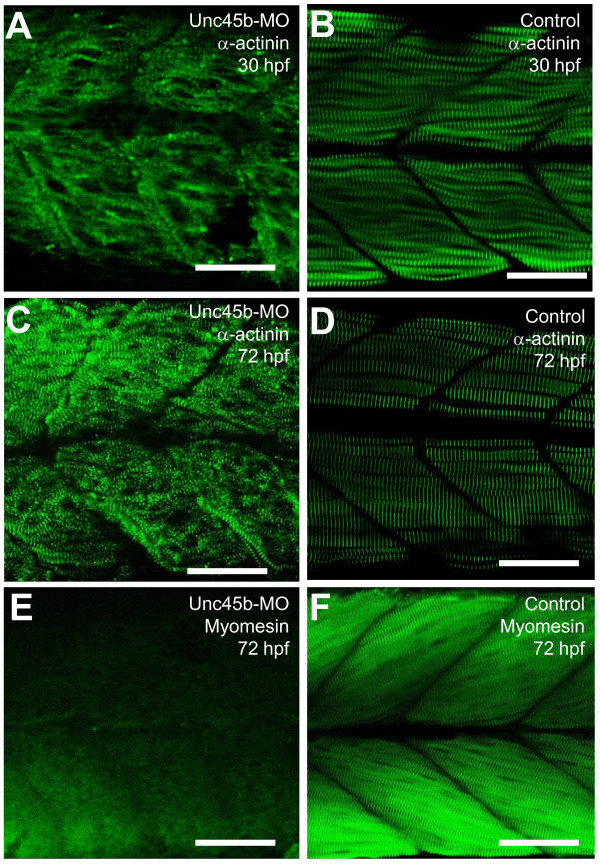
**The effect of *unc-45b *knockdown on α-actinin and myomesin localization in skeletal muscles of zebrafish embryos**. **A-D**. Anti-α-actinin antibody staining shows the Z-disc organization in unc-45b knockdown (A, C) or uninjecetd control (B, D) embryos at 30 (A, B) or 72 (C, D) hpf. **E, F**. Anti-myomesin antibody staining shows the M-line organization in *unc-45b *knockdown (E), or control (F) embryos at 72 hpf. Scale bars = 25 μm

The M- and Z-line structures of sarcomeres are critical for thick and thin filament organization. α-actinin is a protein that has a significant role in anchoring the actin-rich thin filaments to the Z-line. This anchoring is important in maintaining the stability of the sarcomere during muscle contraction. To determine whether Z-line organization was affected in *unc-45b *knockdown zebrafish embryos, we performed immunostaining with the anti-α-actinin antibody in *unc-45b *knockdown zebrafish embryos. As illustrated in Figure 3A, the sarcomeric localization of α-actinin was disrupted in *unc-45b *knockdown embryos at 30 hpf. A significant recovery of α-actinin organization was seen in the knockdown embryos at 72 hpf (Figure 3C), consistent with the partial recovery of myosin accumulation at this stage (Figure 2). The muscle fibers in *unc-45b *morphant embryos, however, meandered throughout the myotome compared to those in wild type embryos (Figure 3D).

Myomesin is a major constituent of the M-line, responsible for anchoring thick filaments within the sarcomere. Given that knockdown of *unc-45b *resulted in disruption of the thick and thin filament organization, the question of a potential effect on myomesin expression and M-line organization arose. To determine the effect of *unc-45b *knockdown on the sarcomeric of localization myomesin, antibody staining for this protein was performed. As illustrated in Figure 3E, knockdown of *unc-45b *resulted in a significant disrupted the sarcomeric localization of myomesin in zebrafish embryos. Very little sarcomeric localization of myomesin could be detected in the *unc-45b *knockdown embryos (Figure 3E). This is in contrast to the effect observed on the Z-line, suggesting that *unc-45b *knockdown may have a more dramatic disruptive effect on M-lines than Z-lines. This is consistent with the fact that M-lines are primary anchoring sites for myosin thick filaments.

### 3. Over-expression of Unc-45b disrupted myosin thick filament organization

**Figure 4:**
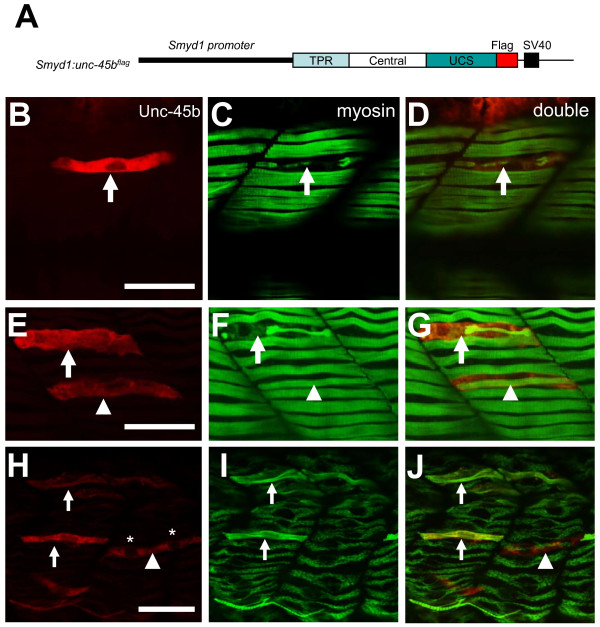
**Overexpression of Unc-45b resulted in defective thick filament organization**. A. **The **DNA construct expressing a flag-tagged Unc-45b under the control of *smyd1 *promoter was injected into zebrafish embryos. The effect on thick filament organization was analyzed by double antibody staining at 30 hpf. **B-D**. Double staining with anti-flag (red) and anti-myosin (F59, green) antibodies shows the expression of flag-tagged *Unc-45b *(B) in a single fiber and its effect on myosin thick filament organization (C). D represents the merged image of B and C showing that the myofibril defect is restricted to the myofiber expressing the flag-tagged Unc-45b. Fifty six myofibers from 40 embryos were analyzed. 70% of the myofiber showed the disorganized thick filaments. **E-G**. Double staining shows the expression of flag-tagged *Unc-45b *in two myofiber with different phenotypes on myosin thick filament organization. One myofiber exhibited disorganized thick filament (Arrow). In contrast, another myofiber exhibited normal thick filament organization (arrow head). However, this fiber appeared to be skinnier compared with its neighbors without the ectopic *Unc-45b *expression. **H-J**. Double staining shows the rescue of thick filament organization in Unc45b knockdown zebrafish embryos co-injected Unc45b-MO with the *smyd1:unc45b^flag ^*DNA construct. Myofibers expressing the flag-tagged Unc45b (H) exhibited normal thick filament organization (I, J). The fast fiber with two nuclei (*) is indicated by the arrow head. F59 does not label myosin expressed in fast muscles. Thirty five slow myofibers from 29 embryos were analyzed. 100% of the slow myofiber expressing the flag-tagged Unc45b (H) exhibited normal thick filament organization. Scale bars = 15 μm.

It has been reported that Unc-45 chaperone mediates sarcomere assembly involving myosin degradation in *C. elegans *[12]. Unc-45 protein levels are tightly regulated by ubiquitylation in *C. elegans *[17]. Down-regulation or overexpression of Unc-45 results in defective myosin folding and assembly in body wall muscles of *C. elegans *[12]. However, the effect of Unc-45b overexpression has not been analyzed in vertebrate muscles. To determine if overexpression of Unc-45b in zebrafish embryos was detrimental to the developing muscle fiber, we decided to express Unc-45b in zebrafish embryos using the transient expression approach via DNA microinjection. A DNA construct expressing a FLAG-tagged full-length zebrafish Unc-45b was generated (Figure 4A). The construct was targeted for muscle-specific expression using the zebrafish muscle-specific *smyd1 *promoter [21]. The *smyd1b:unc-45b^flag ^*DNA construct was microinjected into zebrafish embryos at 1-2 cell stages. As expected, a muscle-specific mosaic pattern of expression was detected in injected embryos by anti-FLAG antibody staining (Figure 4B).

To determine whether ectopic expression of Unc-45b^flag ^affected myosin thick filament organization, the injected embryos were analyzed by double immunostaining using anti-FLAG and anti-myosin (F59) antibodies. As illustrated in Figure 4C-D, ectopic expression of the flag-tagged Unc-45b resulted in disruption of the myosin filament organization in slow muscle fibers. This phenotype was observed in 70% (n = 56) of the slow myofibers expressing the flag-tagged Unc-45b. The other 30% of slow fibers expressing the Unc-45b^flag ^did not show the complete disruption of thick filament organization. However, these fibers appeared to be smaller when compared with their neighboring myofibers that did not express the Unc-45b^flag ^(Figure 4E-G). Interestingly, these two types of phenotype could appear in the same fish embryo as shown in Figure 4E-G. Moreover, there appears to be a correlation with respect to the levels of Unc-45b^flag ^expression. Myofibers with a stronger Unc-45b^flag ^immunofluoresence staining showed a dramatic disruptive phenotype (Figure 4E-G). In contrast, myofibers with a weak anti-Unc-45b^flag ^staining showed the smaller fiber phenotype with the narrower myosin thick filaments (Figure 4E-G).

To confirm the specificity of the phenotye and to rule out the possibility that the flag-tagged Unc-45b acts as a dominant negative, we performed the rescue experiment by co-injecting the Unc45b ATG-MO with the Unc-45b expression construct. The data showed that the thick filament defect from Unc-45b knockdown could be rescued by expression of the Unc45b-flag transgene (Figure 4H-J). Such data indicate that the Unc-45b-flag minigene could actually substitute the function of Unc-45b, and the thick filament defect observed in Unc-45b overexpressing fibers was unlikely due to a dominant negative effect. To further exclude the possibility that disruption of myosin organization could occur regardless of which protein was overexpressed, we performed a series of control experiments using gene constructs expressing EGFP or Hsp90α1 proteins (additional file 1: supplemental Figure 1). The results showed that expression of EGFP or Hsp90α1 in myofibers had no effect on myosin expression and thick filament organization (additional file 1: supplemental Figure 1). Together, these data affirm the specificity of the Unc-45b overexpression phenotype.

### 4. Deletion of the C-terminal UCS domain abolished the disruptive effect of Unc-45b overexpression on thick filament organization

**Figure 5:**
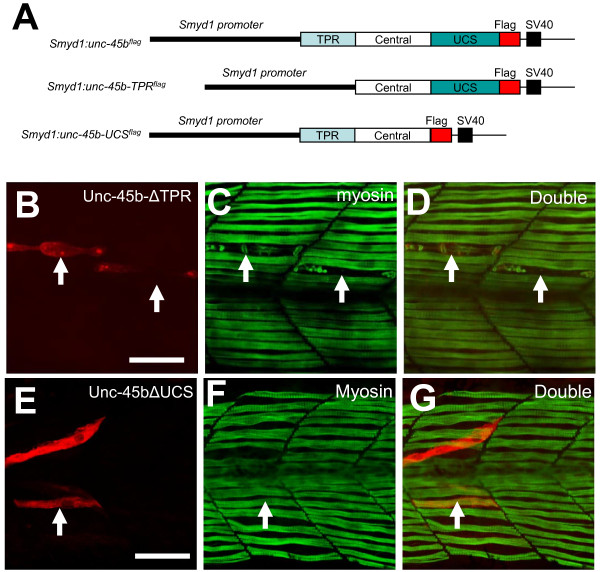
**Deletion of the N-terminal TPR domain had no effect on the disruptive activity of Unc-45b on myosin thick filament organization, whereas deletion of the C-terminal UCS domain abolished the disruptive effect of Unc-45b overexpression**. **A**. DNA constructs, *smyd1:unc-45bΔTPR^flag ^*and *smyd1:unc-45bΔUCS^flag^*, were generated as minigenes expressing Unc-45b mutant proteins without the N-terminal TPR domain (amino acids 1-119), or the C-terminal UCS domain (amino acids 537-934). A flag-tag was fused in frame at the C-terminus for monitoring gene expression. The DNA construct was injected into zebrafish embryos. The effect on thick, thin filament and Z-line organization was analyzed by double staining using anti-flag (red) and anti-myosin (F59, green) antibodies at 30 hpf. **B-D**. Expression of flag-tagged TPR deletion mutant of Unc-45b (B) in fiber disrupts thick filament organization (C). D represents the merged image of B and C. Forty two myofibers from 36 embryos were analyzed. 75% of the myofiber showed the disorganized thick filaments. **E-G**. Deletion of the C-terminal UCS domain abolished the disruptive effect of Unc-45b overexpression. Myofiber expressing the UCS deletion mutant (E) exhibited normal thick filament organization (F). G represents the merged image of E and F. Twenty nine myofibers from 25 embryos were analyzed. None of the myofiber showed the disorganized thick filaments. Scale bars = 25 μm.

Unc-45b is comprised of three major domains: an N-terminal TPR domain, which interacts with Hsp90α, a central domain of unknown function, and a C-terminal UCS domain that interacts with the myosin head. In an attempt to better ascertain the significance of each domain in sarcomere assembly, we generated two mutant constructs missing either the TPR or UCS domain of Unc-45b (Figure 5A). These constructs were expressed in zebrafish embryonic myofibers directed by the muscle-specific *smyd1 *promoter [21]. The results showed that deletion of the N-terminal TPR domain (ΔTPR) had no effect on the disruptive activity of Unc-45b on thick filament organization (Figure 5B-D). Myofibers expressing the Unc-45bΔTPR mutant construct showed a significant disruption of thick filament organization (Figure 5C, D). The phenotype was very similar to that observed in myofibers overexpressing the full-length Unc-45b protein (Figure 4B-D). Taking into consideration that the TPR domain is required for binding with Hsp90α[6], these data indicate that interaction with Hsp90α1 is not required for disruption of myosin thick filament organization from Unc-45 overexpression.

In contrast to the TPR mutant, the C-terminal UCS domain appeared to be involved in the disruptive effect of Unc-45b on myosin organization. Deletion of the C-terminal UCS domain abolished the disruptive effect of Unc-45b overexpression on thick filament organization (Figure 5E-G). As seen in Figure 5G, myofibers overexpressing the Unc-45bΔUCS mutant construct appeared normal with organized myosin thick filaments. Taking into consideration that the UCS domain is required for interaction with myosin [11,16], these data indicate that Unc-45b interaction with myosin may be involved in disruption of myosin organization resulting from the Unc-45b overexpression.

## Discussion

In this study, we showed that proper levels of Unc-45b expression are critical for organized assembly of thick filaments. Both knockdown and overexpression of Unc-45b resulted in defective myosin thick filament organization. In addition, knockdown of *unc-45b *disrupted the organization of thin filaments, Z- and M-lines. We further showed that the disruption of thick filament organization by Unc-45b overexpression depends on the UCS domain at the C-terminus of Unc-45b, which is required for binding with myosin. Deletion of the UCS domain abolished the disruptive effect by Unc-45b on the myosin thick filament assembly. In contrast, deletion of the TPR domain required for interaction with Hsp90α had no effect. Together, these studies indicate that expression levels of Unc-45b must be precisely regulated for normal myofibril organization in skeletal muscles of zebrafish embryos.

### Unc-45b is required for myosin expression and sarcomere assembly

Our data indicate that Unc-45b is essential for sarcomere organization in zebrafish skeletal muscles. Knockdown of *unc-45b *through the use of morpholino oligos had a profoundly negative impact on myosin and actin protein accumulation as well as their assembly into sarcomeres. The defective thick filament organization from *unc-45b *knockdown is not surprising, given the known function of Unc-45b as a myosin chaperone and Hsp90α co-chaperone. There data are consistent with previous reports that Unc-45 is co-localized with myosin heavy chain in *C. elegans *and zebrafish muscle cells [22,23]. Moreover, Unc-45b forms a cytosolic complex with Hsp90 and interacts with the myosin motor domain, facilitating appropriate folding [3].

We demonstrated in this study that knockdown of *unc-45b *had a dramatic impact on sarcomeric localization of myomesin. Myomesin is a major component of the M-line, responsible for anchoring myosin thick filaments [24]. Myomesin protein appears to have spring-like capacities, similar to titin [25]. Its N-terminal interacts with myosin, while other regions interact with titin and MM-CK [26]. Very little or no sarcomeric localization of myomesin could be detected in the *unc-45b *knockdown zebrafish embryos. Because the function of the M-line is the maintenance of the hexagonal thick filament array in the A-band, it is expected that disruption of thick filament assembly and organization by Unc45b knockdown could block sarcoemric localization of myomesin to the M-line and lead to M-line disorganization. This is consistent with the Unc45b knockdown phenotype from this study.

### Excessive levels of Unc-45b are detrimental to sarcomere organization

Our studies showed that overexpression of Unc-45b resulted in defective myosin thick filament organization in myofibers of zebrafish embryos. The defective thick filament organization in myofibers overexpressing Unc-45b resembles the phenotype from Unc-45 overexpression in the nematode *C. elegans*. In *C. elegans*, overexpression of Unc-45 resulted in decreased thick filament assembly, decreased accumulation of body wall myosins, and mild paralysis [12]. It has been reported that a minigene expressing an Unc-45b-GFP fusion protein, when injected into zebrafish embryos, had no obvious defects in thick filament organization [23]. The discrepancy of the phenotypes could result from the different levels of Unc-45b expression observed in these two experiments. In this study, we used the zebrafish *smyd1 *promoter to drive the expression of Unc-45b, in contrast to the *cmv *promoter used to direct the expression of the Unc-45b-GFP fusion protein [23]. The *cmv *promoter does not possess such skeletal muscle specificity. On the other hand, the zebrafish *smyd1 *promoter is a very strong and muscle-specific promoter, likely leading to high levels of Unc-45b protein expression in muscle cells.

It has been clearly demonstrated in *C. elegans *that the myofibril defects could only be detected in transgenic worm that express high levels of Unc-45 [12,17]. A modest increase in Unc-45 expression had minimal effect on myofibril organization [17], suggesting that the disruptive effects from Unc-45 expression is dose-dependent. Consistent with studies in *C. elegans*, we showed that there appears to be a correlation between the levels of Unc45b overexpression and thick filament disruption (Figure 4). Myofibers with a stronger Unc-45b^flag ^immunofluoresence staining showed a dramatic disrupted phenotype with very little myosin thick filaments (Figure 4E, G). In contrast, myofibers with a weak anti-Unc-45b^flag ^staining showed the smaller myofiber phenotype with the narrower myosin thick filaments (Figure 4E, G, and data not shown). Together, these data indicate that the disruptive effect of Unc-45b is dose dependent, supporting the idea that the thick filament defect was caused by Unc-45b overexpression.

It has been shown that the ubiquitin-proteasome system plays a critical role in regulating the proper levels of Unc-45b expression ensuring normal muscle development [2,17]. Mutations in human p97, an ubiquitin-selective chaperone, abrogate Unc-45 degradation and result in hereditary inclusion body myopathy [18,19]. Collectively, these studies indicate that the precise regulation of Unc-45 expression is essential for normal myofibril organization and function in both invertebrates and vertebrates. Excessive levels of Unc-45b are detrimental to myofibril organization.

The molecular mechanism by which overexpression of Unc-45b disrupts myosin thick filaments is not clear. It has been shown that excessive levels of Unc-45 specifically target myofibrillar myosins for proteasome degradation in *C. elegans *[12]. Through pull-down assays, Landsverk and colleagues were able to detect the increased interaction between the ubiquitin-proteasome system (UPS) and myosin in Unc-45 overexpressing *C. elegans *[12]. Several models have been proposed to explain the effects of Unc-45 overexpression on myosin folding, thick filament assembly and protein degradation. It has been suggested that excessive Unc-45b may result in the super-stabilization of individual myosin units, slowing down their assembly into sarcomeres and leading to increased protein degradation by the ubiquitin-proteasome system [4]. Others proposed that these excessive levels of Unc-45b may result in defective myosin thick filament organization; driving assembled thick filaments back towards an unassembled state, again leaving the individual myosin units vulnerable to aggregation and subsequent degradation by the UPS [2,12].

Considering that different mechanisms may be behind the disruption of thick filament organization by Unc-45b overexpression and knockdown, it remains to be determined whether overexpression of Unc-5b could disrupt the organization of other sarcomeric structures, including the thin filaments, M and Z-lines. A comparative analysis of knockdown and overexpressiosn phenotypes could provide important insight into the temporal sequence of events involved in sarcomere assembly and maintenance during muscle cell differentiation. It has been reported that the first sarcomeric components to appear in zebrafish skeletal muscles are I-Z-I bodies, consisting of small puncta of α-actinin flanked on both sides by actin filaments [8,27]. As muscle cell differentiation proceeds, the punctate pattern of α-actinin in the Z-bands become aligned and uniformly stained Z lines. Myosin thick filament appears to be critical for the Z-line alignment. Knockdown of myosin expression in zebrafish embryos resulted in defective alignment of Z-lines without any effect on the initial I-Z-I body formation [28]. This indicates that the initial I-Z-I formation is independent of myosin thick filament assembly, consistent with the earlier appearance of I-Z-I bodies compared with the thick filaments.

### Deletion of the C-terminal UCS domain abolished the disruptive effect of Unc-45b overexpression on thick filament organization

Our data indicate that deletion of the C-terminal UCS domain abolished the disruptive effect of Unc-45b overexpression on thick filament organization. Muscle fibers expressing a UCS deletion mutant exhibited normal thick filament organization. UCS domains are found in many proteins involved in interactions with myosin [29]. The UCS domain appears to ensure proper folding of myosin heads such that they can perform their ATP-dependent actin-based motor functions [16,29]. Mutations in the conserved regions of the Unc-45 UCS domain result in reduced numbers of myofilaments with severe disorganization in *C. elegans *[30]. Collectively, these data indicate that interaction with myosin via the UCS domain is critical for myosin folding and assembly under normal conditions and is involved in disruption of myosin thick filament organization from Unc-45b overexpression. However, we could not rule out the possibility that the truncated protein is a new entity and may have no activity because it is unstable or improperly folded.

We have demonstrated that, unlike the UCS domain, deletion of the N-terminal TPR domain had no effect on the disruption of myosin thick filament organization. Similar to the full-length Unc-45b, overexpression of the TPR deletion mutant resulted in decreased myosin expression and disruption of thick filament organization. It has been shown that the N-terminal TPR repeats are involved in interactions with Hsp90α, a known myosin chaperone. Deletion of the TPR domain abolished Unc-45 interaction with Hsp90α in *C. elegans *[16]. Despite this lack of interaction with Hsp90α, we showed that overexpression of the N-terminal truncated Unc-45b severely impeded myosin organization in zebrafish skeletal muscles. These data indicate that interaction with Hsp90α1 is not essential for the muscle phenotypes from Unc-45b overexpression. Because the TPR deletion mutant contains the UCS myosin binding domain, it could compete with Unc-45b for myosin binding and thus act as a dominant negative that blocks the function of endogenous Unc45b. However, it should be noted that without a structure for these mutant proteins it is difficult to predict whether the TPR and UCS homology regions correspond to discrete folding domains.

### Hypothetic models

Our data, together with previous findings in *C. elegans *and zebrafish, allow us to formulate several hypotheses relating to the significance of critical levels of Unc-45b in sarcomere assembly. Under normal conditions (proper levels of Unc-45b expression), Unc-45b is required for myosin folding and assembly. This function requires Unc-45b interaction with Hsp90α1. Loss of Unc-45b or Hsp90α1 function results in a failure in folding of the myosin head domain, leading to significant reduction of myosin accumulation and disruption of thick filament formation. Without thick filaments, other aspects of sarcomere assembly including development of the thin filaments, Z-discs, and M-lines does not occur normally.

Just as insufficient Unc-45b prevents sarcomere assembly, excessive levels of Unc-45b is detrimental to myofibril organization. Overexpression of Unc-45b results in defective thick filament organization. Two alternative possibilities present themselves as explanations for the lack of thick filament organization in Unc-45b overexpressing myofibers. One hypothesis is that normal myosin folding occurs in Unc-45b overexpressing myofibers. However, individual myosin units associated with excessive Unc-45b are unable to congregate and form thick filaments. These myosin units become vulnerable to degradation by the UPS. A second hypothesis is that myosin units are able to assemble into thick filaments in the presence of high levels of Unc-45b. However, the excess Unc-45b remains associated with myosin units for an extended period of time, preventing appropriate interactions with M-line protein or anchoring proteins such as titin. These thick filaments are rendered unstable and easy targets for degradation through the UPS. In either case, the disruptive effect of Unc-45b does not require interaction with Hsp90α1, but does require interaction with myosin.

## Conclusion

In summary, we have carried out the knockdown and overexpression studies to analyze Unc-45b function in myofibril organization in zebrafish embryos. We have demonstrated that proper levels of Unc-45b expression are critical for normal thick filament assembly. Both knockdown and overexpression of Unc-45b resulted in defective myosin thick filament organization. Together, these studies demonstrate that levels of Unc-45b expression must be precisely regulated during myofibrillogenesis. Low or high levels of Unc-45b expression could result in adverse effects on myofibril assembly.

## Methods

### Synthesis of morpholino oligos

The expression of Unc-45b and Hsp90α1 were individually knocked down using morpholino-modified antisense oligos that block protein translation. These morpholinos were generated as previously described [7,10]. Both morpholino oligos were suspended in 1× Danieau buffer [31] to a final concentration of 0.5 mM along with 0.1% phenol red to allow for easier visualization during injection. Standard control morpholino from Gene Tools was used as control. Sequences for the Unc-45b and Hsp90α1 morpholino oligos were as follows:

Unc-45b ATG-MO: 5' -ATCTCCAATTTCTCCCATCGTCATT- 3'

Hsp90α1 ATG-MO: 5'-CGACTTCTCAGGCATCTTGCTGTGT-3'

### DNA constructs

DNA constructs, *smyd1:unc-45b^flag^*, *smyd1:unc-45bΔTPR^flag^*, and *smyd1:unc 45bΔUCS^flag^*, were generated as minigenes expressing a flag-tagged zebrafish full-length Unc-45b, a mutant Unc-45b without the TPR domain (amino acids 120-934), or a mutant Unc-45b without the C-terminal UCS domain (amino acids 1-536). The muscle-specific expression of these constructs was obtained using the muscle-specific *smyd1 *promoter [21]. The DNA constructs were suspended in distilled water at 50ng/μl along with 0.1% phenol red.

To construct the *smyd1:unc-45b^flag ^*minigene, a full-length *unc-45b *coding region was amplified from the 24 hpf zebrafish embryos by RT-PCR using unc-45b-5'ER1 and unc-45b-3' primers together with the advantage 2 DNA polymerase (Clontech). The PCR product was cloned into the pGEM-T Easy Vector (Promega). To place a flag-tag sequence at the C-terminus of the protein, a 3' primer was made with a flag-tag coding sequence in frame at the end of the Unc-45b C-terminus. The Unc-45b was amplified using the 5' ATG primer together with the 3'-flag primer with pfu DNA polymerase. The PCR product was cloned into the pBluescript sk vector SmaI site. The DNA insert was then released from the plasmid by BamHI digestion. The insert containing the flag-tagged Unc-45b was cloned after the zebrafish muscle-specific *smyd1 *promoter at the BamHI site [21]. A SV40 polyA sequence was included in the expression vector. The resulting plasmid was named *smyd1:unc-45b^flag^*.

The mutant constructs expressing a TPR or UCS domain deleted Unc-45b were constructed using PCR. The TPR domain deletion was carried out using PCR with the unc-45b-ΔTPR5' and unc-45b-3'flag primers. The PCR product was cloned after the *smyd1 *promoter to generate the expression construct *smyd1:unc-45bΔTPR^flag^*. Similarly, DNA coding sequence without the UCS domain was amplified by PCR using the unc-45b-5'ER1 and unc-45b-5'ER1 primers. The PCR product was cloned after the *smyd1 *promoter to generate construct *smyd1:unc-45bΔUCS^flag^*.

unc-45b-5'ER1: gaattcaatgacgatgggagaaattgg

unc-45b-3': gctctggctaagatcgctgctgt

unc-45b-3'flag: ctacttgtcatcgtcatctttataatcgttggaaaagggctttataagtcc

unc-45b-ΔTPR5': Atggagaccctcaggagacttggagct

unc-45b-5'ER1: ctacttgtcatcgtcgtccttgtagtcaccttcaatggcccactttctggt

The plasmids *smyd1:egfp *and *smyd1:hsp90a1^myc ^*were used as controls. Constructions of these plasmids were described as previously reported [7,21].

### Microinjection of DNA constructs and morpholino oligos

DNA constructs were dissolved in water at a concentration of 50 ng/μl along with phenol red (0.1%). Small volumes (1-2 nl) of the individual DNA constructs (*smyd1:unc-45b^flag^*, *smyd1:unc-45bΔTPR^flag^*, and *smyd1:unc-45bΔUCS^flag^*) were injected into zebrafish embryos at early cell stages of 1 or 2 cells. Embryos were then placed in a 29°C incubator for 24-72 hours in order to facilitate animal growth. Similarly, 1-2 nl of MO solution (0.5 mM) was microinjected into zebrafish embryos at 1-2 cell stages.

### Antibody staining and confocal microscopy

Following injection, embryos were allowed to grow for 24-72 hours post-fertilization prior to dechorionation and fixation in 4% paraformaldehyde in PBS. Immunostaining was carried out using whole mount zebrafish embryos with the following antibodies: anti-α-actinin (clone EA-53, #A7811, Sigma), anti-myomesin (mMaC myomesin B4, DSHB), anti-α-actin (Ac1-20.4.2, Progen), and anti-MyHC for slow muscles (F59, DSHB). F59 monoclonal antibody was originally prepared against chicken fast skeletal muscle myosin [32]. F59 is unusual in that is also reacts beta cardiac myosin, a slow isoform of rats, mice and humans. It was later found that F59 could specifically label slow muscle fibers in zebrafish [33,34]. F59 recognizes myosin heavy chain 1 (myhc1) expressed in slow muscles of zebrafish embryos [28].

Secondary antibodies were FITC or TRITC-conjugates (Sigma). For animals injected with FLAG-tagged constructs, anti-FLAG primary (F7425; Sigma) and subsequent appropriate secondary antibodies were additionally used to detect those muscle fibers expressing the injected DNA. The embryos were photographed under an upright microscope (Zeiss, Oberkochen, Germany) equipped with a confocal image analyzer (BIO-RAD Radiance 2100 Imaging Systems, Hercules, CA).

### Western blot analysis

Following injection with either Unc-45b or Hsp-90α1 morpholino oligos, embryos were left to develop between 24 and 72 hours post-fertilization. Specimens were then prepared for a Western blot through homogenization in loading buffer (0.125 M Tris-HCl, pH 6.8; 4% SDS; 20% glycerol; 0.2 M DTT; 0.02% bromophenol blue in distilled water) along with an additional 0.1 mM DTT and 1 mM PMSF (both as protease inhibitors). Protein samples were run in a 7.5% polyacrylamide gel at 30-32 mAmps for protein separation. Proteins were then transferred onto a nitrocellulose membrane in transfer buffer (20% methanol, 25 mM Tris, 192 mM glycine, 0.1% SDS in distilled water). Actin and γ-tubulin transfer occurred at 400 mAmp for 1 hour. Myosin transfer occurred at 400 mAmp for 1 hour, followed by an additional 1.5 hours at 450 mAmp. Transfer membranes were stained with overall myosin heavy chain specific-IgG (MF-20, 1:2000; DSHB), actin specific-IgM (JLA-20, 1:2000), and γ-tubulin specific-IgG (1:5000; Cat# T6557, Sigma-Aldrich, St. Louis, MO) antibodies followed by exposure to appropriate HRP-linked secondary antibodies (IgG 1:2000, Cell Signaling Technology, Beverly, MA; IgM 1:5000, Sigma-Aldrich, St. Louis, MO). Pierce ECL Western Blotting Substrate (Thermo Scientific, Rockford, IL) was used for detecting HRP-conjugated bound secondary antibodies. The volume equivalent of three embryos was used at each time stage analyzed. γ-tubulin was used as a loading control.

## List of abbreviations

Hsp90α1: (heat shock protein 90 alpha-1); UPS: (ubiquitin-proteasome system); TPR: (tetratricopeptide repeat), UCS: (Unc-45/CRO1/She4p).

## Authors' contributions

EB carried out the functional analyses of Unc-45b in zebrafish via knockdown and over-expression approaches. EB drafted the manuscript. PZ designed and constructed the minigenes expressing the full length or mutant Unc-45b proteins used in the study. SD conceived of the study, participated in its design and coordination and helped write the manuscript. All authors read and approved the final manuscript.

## Supplementary Material

Additional file 1 **Figure 1 (supplement) showed that overexpression of EGFP or Hsp90a1 had not effect on thick filament organization**. A. DNA constructs expressing EGFP or myc-tagged Hsp90a1 in skeletal muscles of zebrafish embryos were directed using the smyd1 promoter. Myosin thick filament organization was analyzed by F59 staining. B-D. Single staining with anti-myosin (F59) antibody (red) shows that expression of GFP (B) has no effect on myosin thick filament organization (C, D). E-G. Double staining shows the expression of myc-tagged Hsp90a1 (E) in a single fiber and its lack of effect on myosin thick filament organization (F, G). G represents the merged image of E and F. Scale bars = 20 mm. Click here for file
