# Supplementary material for: Knockdown and overexpression of Unc-45b result in defective myofibril organization in skeletal muscles of zebrafish embryos
Source: BMC Cell Biol. 2010 Sep 17;11:70. doi: 10.1186/1471-2121-11-70 (PMC2954953; doi:10.1186/1471-2121-11-70)
Supplement: Additional file 1 — Figure 1 (supplement) showed that overexpression of EGFP or Hsp90a1 had not effect on thick filament organization. A. DNA constructs expressing EGFP or myc-tagged Hsp90a1 in skeletal muscles of zebrafish embryos were directed using the smyd1 promoter. Myosin thick filament organization was analyzed by F59 staining. B-D. Single staining with anti-myosin (F59) antibody (red) shows that expression of GFP (B) has no effect on myosin thick filament organization (C, D). E-G. Double staining shows the expression of myc-tagged Hsp90a1 (E) in a single fiber and its lack of effect on myosin thick filament organization (F, G). G represents the merged image of E and F. Scale bars = 20 mm. [file 1471-2121-11-70-S1.PPT]

## Slide 1
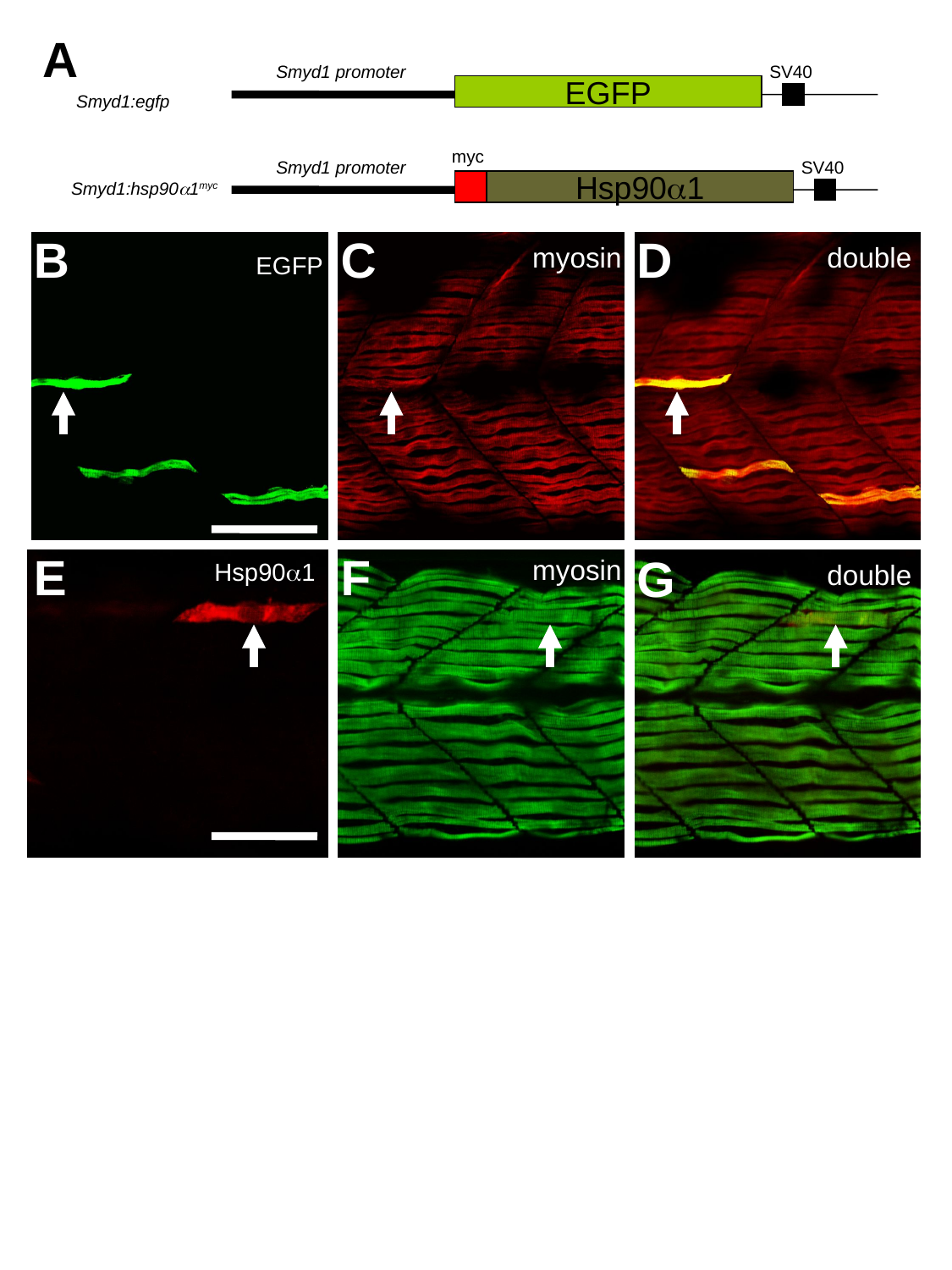

A
Smyd1 promoter
SV40
EGFP
Smyd1:egfp
myc
Smyd1 promoter
SV40
Smyd1:hsp90α1myc
Hsp90α1
B
C
D
myosin
double
EGFP
E
F
G
myosin
Hsp90α1
double
